# Physiological and Intestinal Microbiota Responses to the Feeding Stimulant Dimethyl-β-Propiothetin (DMPT) in Aquatic Animals—A Preliminary Study on *Pontastacus leptodactylus* Fed on a Plant-Based Diet

**DOI:** 10.3390/antiox15060715

**Published:** 2026-06-04

**Authors:** Ying Yan, Ming Li, Yanjie Tang, Xiting Chen, Haibo Jiang, Muzi Zhang, Na Li, Bin Li

**Affiliations:** 1 School of Marine Sciences, Ningbo University, Ningbo 315211, China; 2401130144@nbu.edu.cn (Y.Y.); 2301130097@nbu.edu.cn (Y.T.); 2411130076@nbu.edu.cn (X.C.); 2 College of Animal Science, Guizhou University, Guiyang 550025, China; jhb99412@126.com (H.J.); mzzhang3@gzu.edu.cn (M.Z.); 3 College of Biosystems Engineering and Food Science, Zhejiang University, Hangzhou 310058, China; 4 College of Veterinary Medicine, Xinjiang Agricultural University, Urumqi 830052, China; nali@xjau.edu.cn

**Keywords:** DMPT, plant protein diet, *Pontastacus leptodactylus*, feed intake, antioxidant capacity, intestinal microbiota

## Abstract

The replacement of fishmeal with plant protein is a key strategy for sustainable aquaculture, but reduced feed intake and digestive efficiency remain major constraints. This study evaluated the effects of dietary dimethyl-β-propiothetin (DMPT) supplementation on feed intake, digestive function, antioxidant capacity, and intestinal microbiota in narrow-clawed crayfish (*Pontastacus leptodactylus*) fed an all-plant protein diet. Three isonitrogenous and isolipidic diets were formulated: a plant protein diet (PPD), an animal protein diet (APD), and a PPD supplemented with 0.5% DMPT. After a 4-week feeding trial, results showed that PPD significantly reduced feed intake and digestive enzyme activities compared to APD, whereas DMPT supplementation restored feed intake to a level comparable to APD, maintained growth-related parameters at intermediate levels, and significantly enhanced α-amylase (AMS), lipase (LPS), and trypsin (TPS) activities. Additionally, DMPT markedly improved hepatopancreatic antioxidant capacity, as indicated by increased total antioxidant capacity (T-AOC), glutathione (GSH), catalase (CAT), and superoxide dismutase (SOD) levels, without affecting muscle composition or intestinal morphology. Microbiota analysis revealed that DMPT altered community structure, increased Bacillota abundance, and promoted microbial network stability. Overall, DMPT supplementation effectively mitigates the limitations of plant protein diets and supports the replacement of animal protein in crayfish aquafeeds.

## 1. Introduction

*Pontastacus leptodactylus*, commonly known as the narrow-clawed crayfish, is a freshwater crustacean of considerable economic significance that is extensively distributed throughout Eastern Europe and certain regions of the Middle East [1]. In recent years, its presence in the Irtysh River Basin of Xinjiang, China, has been formally documented, and the species has rapidly attracted increasing market interest [2]. Notably, its market price is approximately 20–25 times higher than that of the invasive red swamp crayfish (*Procambarus clarkia*), further driving the expansion of its artificial aquaculture. Feed constitutes the largest operational cost in aquaculture, typically accounting for 50–70% of total production expenses [3,4]. Among feed ingredients, animal-derived proteins, particularly fishmeal, are the costliest. Consequently, reducing dietary fishmeal inclusion has become a central focus in aquafeed research [5].

Our recent study demonstrated that a plant-based diet represents a feasible nutritional strategy for narrow-clawed crayfish, as no significant adverse effects on intestinal morphology, antioxidant enzyme activities, or survival rate were observed compared with an animal protein-based diet (unpublished). However, complete replacement of animal protein with plant ingredients resulted in reduced feed intake and decreased digestive enzyme activities [6,7]. The limited palatability of plant-based diets has been widely recognized as a major constraint in aquaculture [8,9]. Feeding stimulants are commonly applied to address this issue, such as organic acids in gilthead seabream (*Sparus aurata*) [10], microencapsulated inulin in striped catfish (*Pangasianodon hypophthalmus*) [11], and earthworm extract in Caspian roach (*Rutilus caspicus*) [12]. Therefore, the identification of effective feed additives capable of enhancing feed intake is essential for facilitating the practical application of all-plant protein diets in the commercial culture of narrow-clawed crayfish.

Nowadays, dimethyl-β-propiothetine (DMPT) has emerged as a widely recognized feeding stimulant in aquaculture. Liu et al. [13] illustrated that adding DMPT to a wholly plant protein diet could increase feed intake and digestive function, and further improve the intestinal structural integrity of grass carp (*Ctenopharyngodon idella*). Similar beneficial effects have also been observed in largemouth bass (*Micropterus salmoides*) [14], Pacific white shrimp (*Litopenaeus vannamei*) [15], and red sea bream (*Pagrus major*) [16]. Moreover, dietary DMPT has been reported to enhance antioxidant capacity in abalone (*Haliotis discus hannai*) by regulating the expression of *cu/zn sod*, *nrf2*, and *keap1* in the digestive gland [17], and to improve muscle quality in grass carp through the Nrf2 signaling pathway [18]. However, whether DMPT exerts similar antioxidant effects in crustaceans remains unknown. Moreover, the intestinal microbial community plays a vital part in nutrient digestion, metabolic processes and immune modulation, and its structural composition is highly susceptible to dietary constituents, including chemical signaling molecules such as feeding attractants [19]. Given its promising antioxidant properties, DMPT may also further promote intestinal health by alleviating oxidative stress, thereby supporting a more favorable microbial community [20]. Nevertheless, the interaction between DMPT and the intestinal microbiota of crustaceans remains poorly understood.

Taken together, the current study for the first time evaluated the effects of dietary DMPT supplementation on feed intake, digestive enzyme activities, intestinal morphology, antioxidant status, and intestinal microbial composition of narrow-clawed crayfish fed an all-plant protein diet. The results aim to provide a theoretical reference for formulating animal protein-free diets and supporting healthy commercial culture of this crayfish species.

## 2. Materials and Methods

### 2.1. Experimental Diets, Feeding Regimen, and Sampling

This study developed three isonitrogenous and isolipidic experimental diets as follows: a plant protein-based diet (PPD), an animal protein-based diet (APD), and a PPD supplemented with 0.5% DMPT (DMPT). The diets were processed into pellets with a diameter of 1.5 mm, subsequently dehydrated at 60 °C, and then preserved at −20 °C until utilization. The formulations and proximate compositions of the experimental diets are detailed in Table 1.

Overall, 90 crayfish (initial body weight = 70.58 ± 1.37 g) were randomly assigned to nine tanks (each with a water capacity of 70 L and housing 10 individuals), with three replicate tanks designated for each treatment. Subsequent to a 2-week acclimation period, a 4-week feeding experiment was carried out under regulated environmental conditions, with the following parameters: water temperature maintained at 20 ± 1 °C, dissolved oxygen content ≥ 7.5 mg/L, and pH value stabilized at 7.8 ± 0.4. Feeding activities were carried out twice daily, with two feeding sessions arranged from 08:00 to 09:00 and 20:00 to 21:00. Daily records were made for the feed quantity delivered to each tank. Once the feeding process was finished, unconsumed feed was carefully retrieved from every tank before being dried and weighed. The apparent feed intake was then determined according to the gap between the supplied feed quantity and the dried residual feed. At the initiation and termination of the feeding experiment, all crayfish underwent weight measurement for the purpose of determining growth performance indices and feed utilization traits, encompassing weight gain rate (WGR), specific growth rate (SGR), feed intake (FI), feed conversion ratio (FCR), and survival rate (SR). The formulas were as follows: WGR (%) = 100 × (FBW − IBW)/IBW; SGR (%/day) = 100 × [ln(FBW) − ln(IBW)]/days; FI (g/crayfish) = total apparent feed intake/final number of crayfish; FCR = total apparent feed intake/total wet weight gain; SR (%) = 100 × final number of crayfish/initial number of crayfish, where IBW and FBW represent initial body weight and final body weight, respectively.

After the feeding experiment was completed, two crayfish were selected randomly from each tank for sample collection (*n* = 6 per treatment). They were anesthetized by immersing them in an 80 mg/L diluted MS-222 solution according to Ma et al. [21], prior to dissection for the harvesting of hepatopancreas and intestinal tissues. In addition, four additional crayfish were collected from each tank; their intestinal contents were extracted and pooled into one composite sample (*n* = 3 per treatment) for intestinal microbiota analysis. In the final step, tail muscle tissues of crayfish from each tank were mixed as one composite sample (*n* = 3 per treatment), which was subsequently used to determine muscle composition.

### 2.2. Proximate Composition Analysis

Moisture, crude lipid, crude protein and crude ash of feeds and muscle tissues were quantified via AOAC standard methods [22].

### 2.3. Antioxidant and Digestive Enzyme Activities

Commercial assay kits from Nanjing Jiancheng Bioengineering Institute (Nanjing, China) were adopted to detect the levels or activities of the following biochemical indicators: total antioxidant capacity (T-AOC), superoxide dismutase (SOD), catalase (CAT), glutathione (GSH), malondialdehyde (MDA), α-amylase (AMS), lipase (LPS).

### 2.4. Intestinal Histological Preparation

Pre-cooled PBS was used to rinse the intestinal tissues, which were then fixed with 4% paraformaldehyde followed by dehydration and embedding procedures. Hematoxylin-eosin (HE) staining was performed on 5 μm thick sections prepared from the embedded muscle tissues. A Nikon TS100 microscope (Tokyo, Japan) was utilized to examine the representative tissue sections. Qualitative histological evaluation of the intestine focused on intestinal structural integrity.

### 2.5. 16S rRNA Sequencing and Analysis

The extraction, purification, and verification of total intestinal DNA, as well as primer synthesis targeting the V4–V5 hypervariable region, were commissioned to Shanghai Biozeron Biotechnology Co., Ltd. (Shanghai, China). High-throughput sequencing was performed on the Illumina PE250 platform (Illumina, Inc., San Diego, CA, USA) at this company. Details of the experimental procedures are described in our previously published study [23].

### 2.6. Statistical Analysis

Data normality and homogeneity of variance were first assessed using the Shapiro-Wilk test and Levene’s test, respectively. For data meeting normality and variance homogeneity criteria, one-way ANOVA and Tukey’s post hoc test were utilized for group difference comparison. For data that did not meet the assumptions for parametric analysis, the Kruskal–Wallis test was applied. Statistical significance was set at *p* < 0.05. All results are reported in the form of mean ± standard error of the mean (SEM). For intestinal microbiota analysis, α-diversity indices, including Chao1, Shannon, Simpson, and Pd_faith, were analyzed using the Kruskal–Wallis test. The LEfSe and heat-tree analyses were carried out with the MicrobiomeAnalyst platform (https://www.microbiomeanalyst.ca/, accessed on 10 November 2025), where differences in taxonomic abundance for the heat-tree analysis were determined using the Wilcoxon rank-sum test.

## 3. Results

### 3.1. Growth Performance and Feed Intake

**Figure 1:**
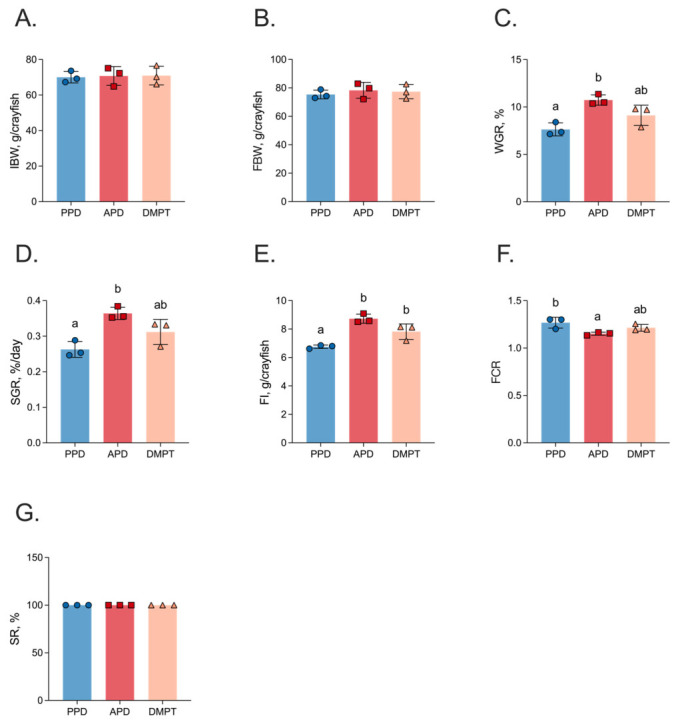
Effects of different protein-source diets on the growth performance and feed intake. (**A**) Initial body weight (IBW); (**B**) final body weight (FBW); (**C**) weight gain rate (WGR); (**D**) specific growth rate (SGR); (**E**) feed intake (FI); (**F**) feed conversion ratio (FCR); and (**G**) survival rate (SR). All data presented as means ± SEM (*n* = 3). Significant differences among groups (*p* < 0.05) were indicated by different letters on the bar charts.

The growth performance and FI of narrow-clawed crayfish are shown in Figure 1. There were no statistically significant differences in IBW and FBW among the three treatment groups (*p* > 0.05; Figure 1A,B). Relative to the PPD group, the APD group had statistically higher WGR and SGR (*p* < 0.05), whereas the DMPT group showed intermediate values and did not differ significantly from either the PPD or APD group (*p* > 0.05; Figure 1C,D). FI levels were statistically higher in the APD and DMPT groups relative to the PPD group (*p* < 0.05; Figure 1E). In addition, FCR was statistically lower in the APD group relative to the PPD group (*p* < 0.05; Figure 1F). The SR hit 100% across every experimental group, with no obvious inter-group disparities (*p* > 0.05; Figure 1G).

### 3.2. Muscle Composition

**Figure 2:**
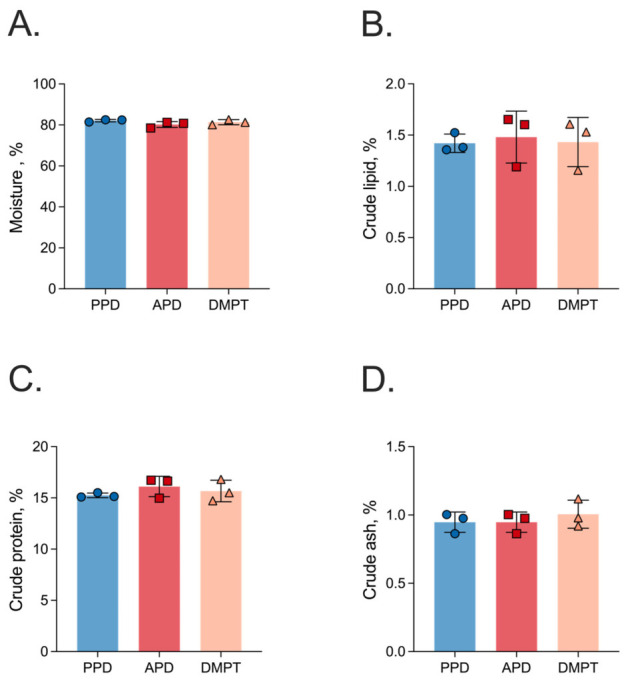
Muscle composition of narrow-clawed crayfish under dietary treatment. (**A**) Moisture; (**B**) crude lipid; (**C**) crude protein; and (**D**) crude ash. All data presented as means ± SEM (*n* = 3).

Figure 2 indicates that the levels of muscle moisture, crude lipid, crude protein and crude ash did not differ significantly between the three treatment groups (*p* > 0.05).

### 3.3. Hepatopancreatic Antioxidant Enzyme Activities

**Figure 3:**
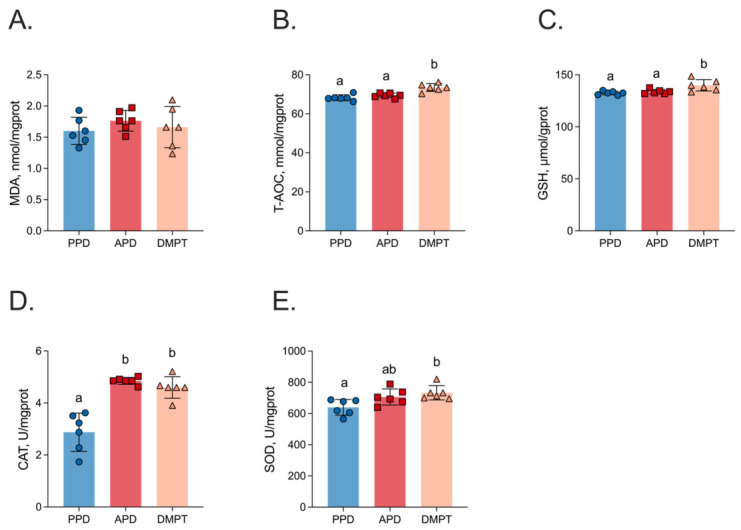
Effects of different protein-source diets on hepatopancreatic antioxidant enzyme activities in narrow-clawed crayfish. (**A**) Malondialdehyde (MDA); (**B**) total antioxidant capacity (T-AOC); (**C**) glutathione (GSH); (**D**) catalase (CAT); and (**E**) superoxide dismutase (SOD). All data presented as means ± SEM (*n* = 6). Significant differences among groups (*p* < 0.05) were indicated by different letters on the bar charts.

No significant differences in hepatopancreatic MDA content were observed among the three treatment groups (*p* > 0.05; Figure 3A). Similarly, there were no statistically significant differences in T-AOC activity (Figure 3B), GSH content (Figure 3C) and SOD activity (Figure 3E) between the PPD and APD groups (*p* > 0.05). Nevertheless, the DMPT group displayed the highest levels of these three indices. Notably, the DMPT group presented obviously higher T-AOC activity and GSH levels relative to the PPD and APD groups (*p* < 0.05), and SOD activity was markedly higher than that in the PPD group (*p* < 0.05). In addition, the APD and DMPT groups had notably higher CAT activity relative to the PPD group (*p* < 0.05; Figure 3D).

### 3.4. Intestinal Histological Structure

**Figure 4:**
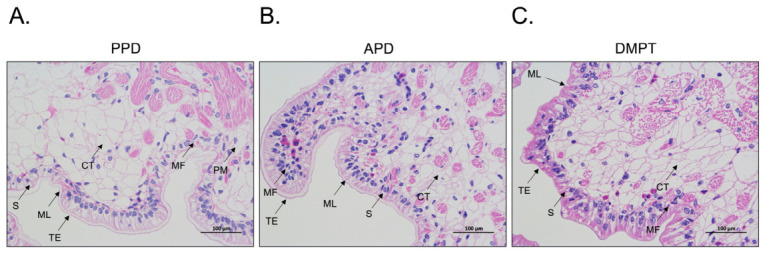
Effects of different protein-source diets on the intestinal histological structure of narrow-clawed crayfish (*n* = 3). (**A**) Intestinal section of crayfish in the PPD group; (**B**) Intestinal section of crayfish in the APD group; and (**C**) Intestinal section of crayfish in the DMPT group. TE: tunica externa; ML: muscular layer; MF: mucosal folds; CT: connective tissue layer; S: epithelial cells; PM: peritrophic membrane.

Histological observations showed that intestinal morphology was well preserved in all three groups, with no obvious pathological lesions (Figure 4). Intestinal villi were structurally intact, regularly arranged, and clearly organized, with no evidence of epithelial damage, inflammatory infiltration, or mucosal destruction.

### 3.5. Intestinal Digestive Enzyme Activities

**Figure 5:**
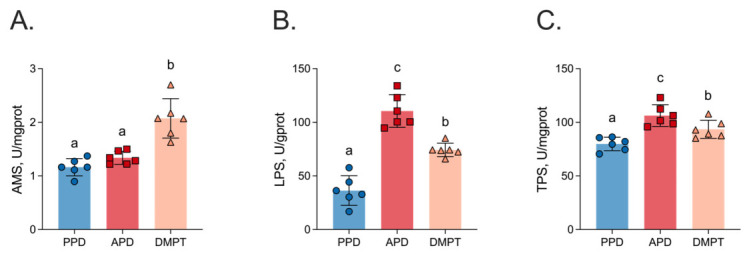
Effects of different protein-source diets on intestinal digestive enzyme activities in narrow-clawed crayfish. (**A**) α-Amylase (AMS); (**B**) lipase (LPS); and (**C**) trypsin (TPS). All data presented as means ± SEM (*n* = 6). Significant differences among groups (*p* < 0.05) were indicated by different letters on the bar charts.

The DMPT group possessed the highest intestinal AMS activity, which was notably higher than that of the remaining two groups (*p* < 0.05; Figure 5A). In addition, the PPD group had notably lower LPS activity relative to the APD group (*p* < 0.05; Figure 5B). Adding DMPT to the diet notably raised LPS activity (*p* < 0.05). Nevertheless, the LPS level in the DMPT group was obviously inferior to that of the APD group (*p* > 0.05). Similarly, the PPD group had notably lower TPS activity relative to the APD group (*p* < 0.05; Figure 5C). DMPT addition markedly elevated TPS activity (*p* < 0.05). Even so, the TPS level of the DMPT group was still obviously inferior to that of the APD group (*p* > 0.05).

### 3.6. Profile of the Intestinal Microbiota

**Figure 6:**
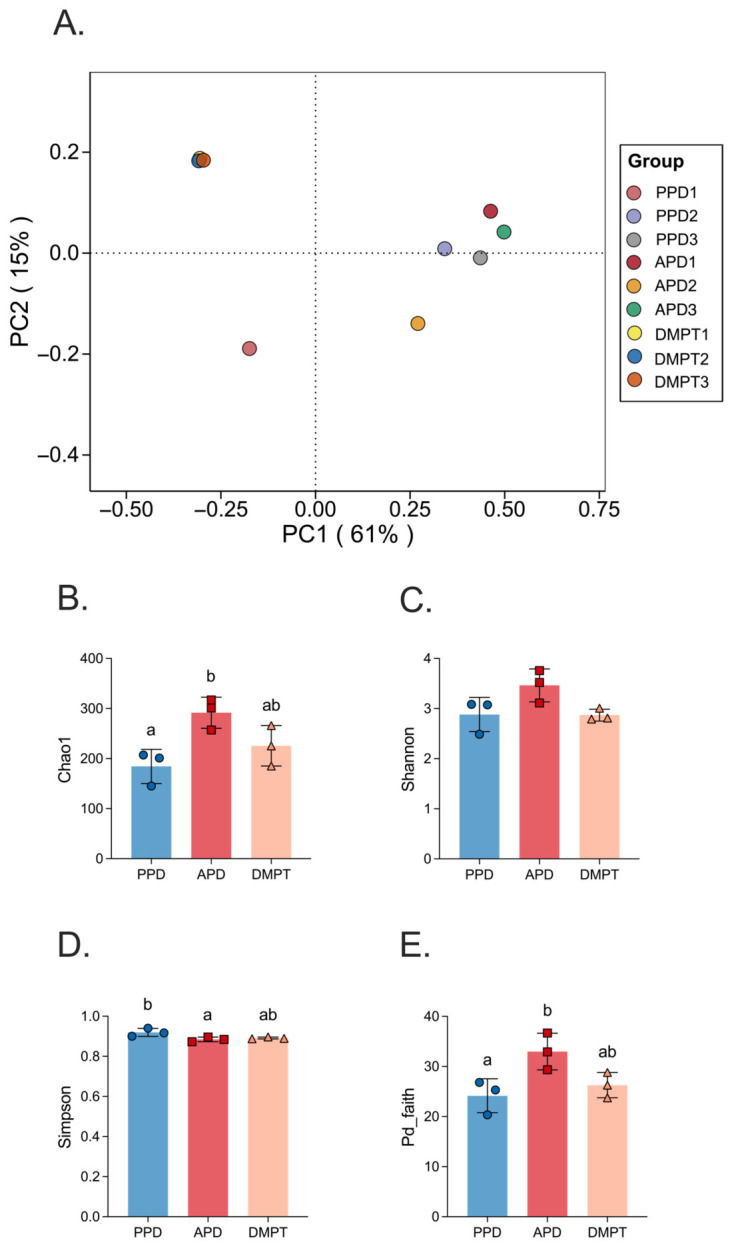
Analysis of α diversity and β diversity of intestinal microbiota (*n* = 3). (**A**). Principal coordinates analysis (PCoA) of the intestinal microbiota; (**B**–**E**). α diversity indexes of intestinal microbiota. Different letters on the bar graphs mark statistically significant differences between groups (*p* < 0.05).

As illustrated in the PCoA score plot (Figure 6A), samples from the PPD and APD groups clustered closely together, while those from the DMPT group were distinctly separated from the other two groups. As shown in Figure 6B–E, the Chao1 and Pd_faith indices were significantly higher in the APD group than in the PPD group (*p* < 0.05), but did not differ significantly from the DMPT group (*p* > 0.05). In addition, the PPD group had a notably higher Simpson index relative to the APD group (*p* < 0.05), while its value was comparable to that of the DMPT group (*p* > 0.05). The Shannon index showed no remarkable differences among the three treatments (*p* > 0.05).

**Figure 7:**
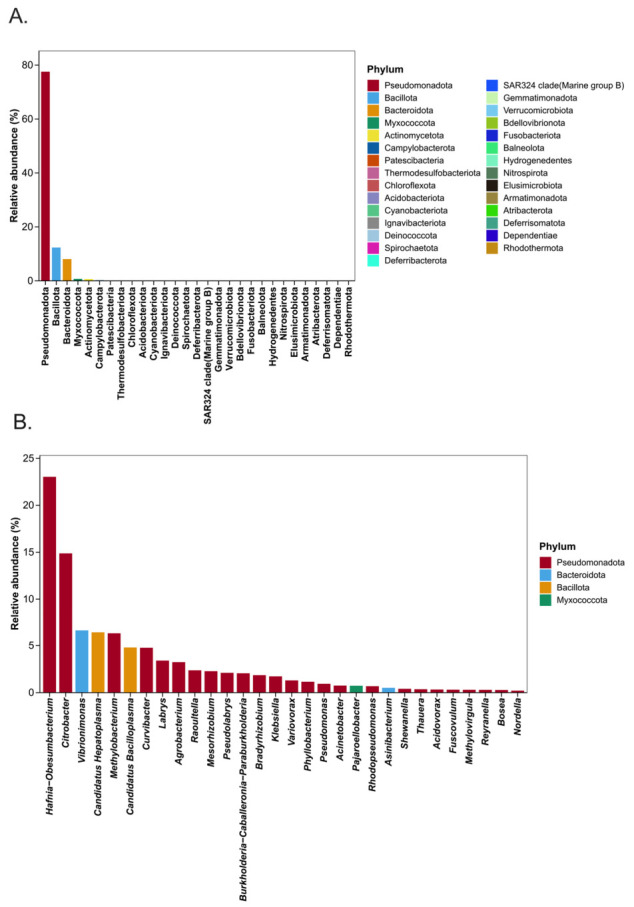
OTUs abundance distribution of all samples (*n* = 3). (**A**) All OTUs annotated at the phylum level; (**B**) The top 30 OTUs annotated at the genus level.

**Figure 8:**
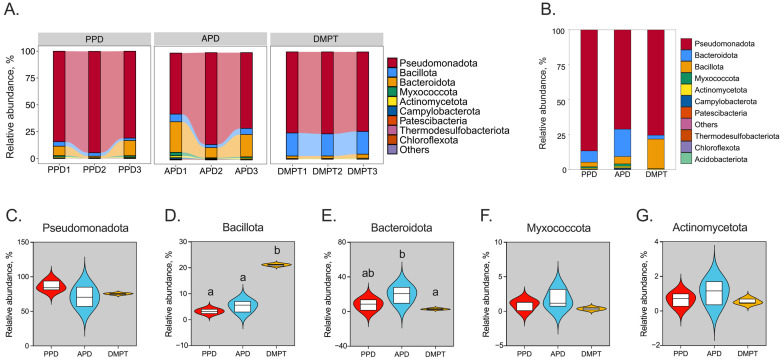
Intestinal microbiota composition of the intestine (*n* = 3). (**A**,**B**). The relative abundance of the top 9 most abundant phyla; (**C**–**G**). Relative abundance of Pseudomonadota, Bacillota, Bacteroidota, Myxococcota, and Actinomycetota, respectively. Different letters on the bar graphs mark statistically significant differences between groups (*p* < 0.05).

As shown in Figure 7A, analysis of OTUs across all samples revealed that Pseudomonadota, Bacillota, and Bacteroidota were the most dominant phyla. At the genus level, the top 10 most abundant genera included *Hafnia-Obesumbacterium*, *Citrobacter*, *Vibrionimonas*, *Candidatus Hepatoplasma*, *Methylobacterium*, *Candidatus Bacilloplasma*, *Curvibacter*, *Labrys*, *Agrobacterium*, and *Raoultella* (Figure 7B). Intestinal microbiota composition of crayfish was further analyzed using stacked bar charts. The results demonstrated that the intestinal microbiota was primarily dominated by Pseudomonadota, Bacillota, and Bacteroidota (Figure 8A,B). The relative abundance of Bacillota was significantly higher in the DMPT group than in the PPD and APD groups (*p* < 0.05; Figure 8D). Meanwhile, the APD group had notably higher Bacteroidota abundance than the DMPT group (*p* < 0.05), while its level was comparable to that of the PPD group (*p* > 0.05; Figure 8E). Furthermore, the three groups showed similar relative abundances of Pseudomonadota, Myxococcota and Actinomycetota (*p* > 0.05; Figure 8C,F–G).

**Figure 9:**
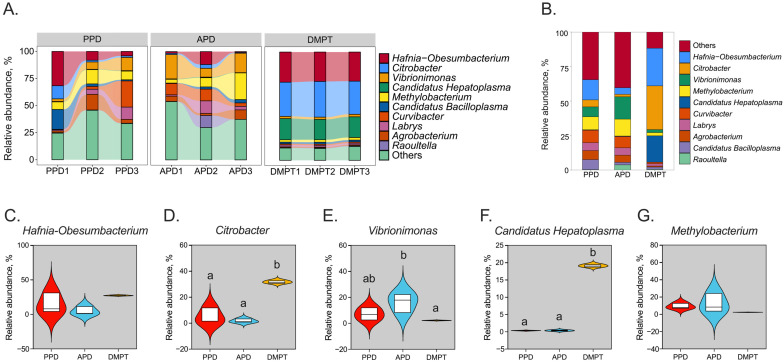
Intestinal microbiota composition of the intestine (*n* = 3). (**A**,**B**). Relative abundance of the top ten predominant microbial genera; (**C**–**G**). Relative abundance of *Hafnia-Obesumbacterium*, *Citrobacter*, *Vibrionimonas*, *Candidatus Hepatoplasma*, and *Actinomycetota*, respectively. Different letters on the bar graphs mark statistically significant differences between groups (*p* < 0.05).

At the genus level, the core intestinal microbiota of crayfish mainly comprised *Hafnia-Obesumbacterium*, *Citrobacter*, *Vibrionimonas*, *Candidatus Hepatoplasma*, and *Methylobacterium* (Figure 9A,B). Compared with the PPD and APD groups, the relative abundances of *Citrobacter* and *Candidatus Hepatoplasma* were significantly increased in the DMPT group (*p* < 0.05; Figure 8D,F). In addition, *Vibrionimonas* had a remarkably higher abundance in the APD treatment than in the DMPT counterpart (*p* < 0.05), while its level was comparable to that of the PPD group (*p* > 0.05; Figure 9E). The abundances of the two genera *Hafnia-Obesumbacterium* and *Methylobacterium* did not differ significantly across all three treatments (*p* > 0.05; Figure 9C,G).

**Figure 10:**
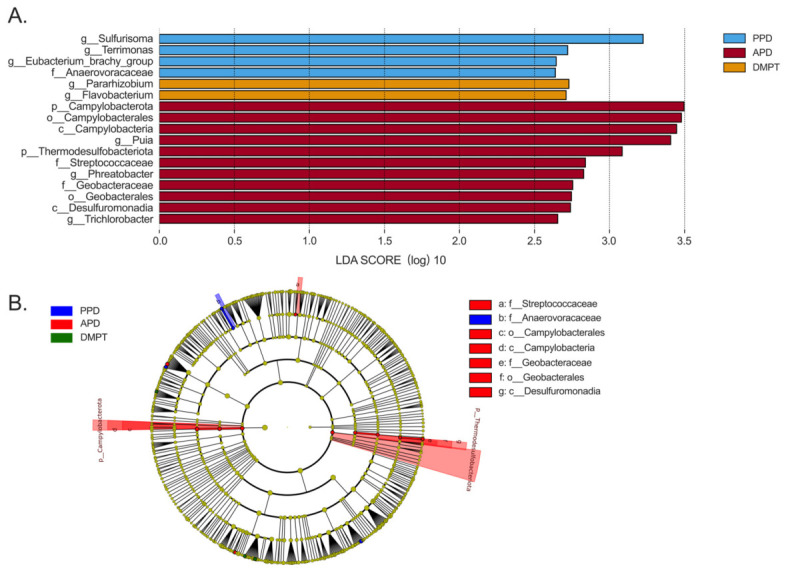
Linear discriminant analysis (LDA) and taxonomic cladogram illustrating taxa with varied relative abundance among groups (*n* = 3). (**A**). Bar graph presenting LDA scores of differential taxonomic taxa. Taxa with statistically significant differences are plotted along the vertical axis, whereas the horizontal axis displays LDA scores (log10); (**B**). The cladogram illustrates the phylogenetic distribution and abundance of core microbial taxa. Each node’s size corresponds to relative abundance.

LEfSe was performed on all OTUs (Figure 10). The results revealed significant differences in *Sulfurisoma*, *Terrimonas*, *Eubacterium_brachy_group*, and Anaerovoracaceae among groups, which were significantly enriched in the PPD group (LDA > 2). In the DMPT group, the relative abundances of *Pararhizobium* and *Flavobacterium* were markedly higher than those in the other groups (LDA > 2). The APD group exhibited the largest number of enriched taxa, including 11 taxa such as Campylobacterota, Campylobacterales, Campylobacteria, *Puia*, and Thermodesulfobacteriota (LDA > 2).

### 3.7. Network Interaction Analysis of Intestinal Microbiota

**Figure 11:**
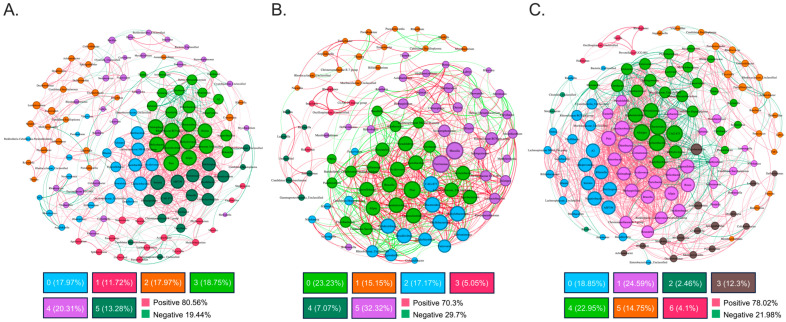
Spearman correlation analysis of intestinal microbiota. (**A**) PPD vs. APD; (**B**) PPD vs. DMPT; and (**C**) APD vs. DMPT. Thick red lines stand for stronger positive correlations, while thick green lines denote stronger negative correlations. Node colors are assigned by modular classes, and the size of each node indicates its intermediate degree centrality.

As shown in Figure 11A, the genus-level co-occurrence network of intestinal microbiota between the PPD and APD groups was clustered into 6 modularity classes. The analysis indicated that 80.56% of the correlations were positive, whereas 19.44% were negative. As displayed in Figure 11B, the corresponding network between the PPD and DMPT groups was also clustered into 6 modularity classes, with 70.3% positive correlations and 29.7% negative correlations. As depicted in Figure 11C, the network between the APD and DMPT groups was clustered into 7 modularity classes, among which 78.02% were positive correlations and 21.98% were negative correlations.

## 4. Discussion

The sustainable development of aquaculture is increasingly constrained by the scarcity and high cost of fishmeal, a key component of traditional aquafeeds [24]. Replacing fishmeal with plant-derived proteins has emerged as a promising strategy to mitigate this challenge [25], and our preliminary study confirmed the feasibility of all-plant protein diets (soybean meal, corn gluten meal, and soy protein concentrate) for narrow-clawed crayfish without compromising overall health. However, the reduced feed intake and digestive enzyme activities observed in crayfish fed such diets highlight a critical bottleneck for the practical application of plant-based aquafeeds, which is consistent with findings in other aquatic species [26,27]. In this context, the present study focused on DMPT, a well-recognized feeding stimulant, to explore its potential to improve the nutritional value of all-plant protein diets for narrow-clawed crayfish, with insights into feed intake, digestive function, antioxidant capacity, and intestinal microbiota dynamics.

Feed intake is a primary determinant of nutrient utilization and growth performance in aquatic animals, and its reduction in plant-based diet-fed crayfish is likely attributed to the suboptimal palatability of plant ingredients [28]. The current results demonstrated that dietary DMPT supplementation significantly enhanced feed intake, which aligns with previous studies on grass carp [13] and large yellow croaker (*Larimichthys crocea*) [29]. This stimulatory effect of DMPT may be related to its structural similarity to natural osmolytes, which can activate chemosensory receptors in the oral cavity and gastrointestinal tract of crustaceans, thereby triggering feeding behavior [29,30]. In addition to FI, growth performance and FCR were further evaluated in the present study. No significant differences were observed in IBW, FBW, or SR among the three groups, indicating that the dietary treatments did not adversely affect short-term growth status or survival of narrow-clawed crayfish. The APD group showed significantly higher WGR and SGR and a lower FCR than the PPD group, reflecting better growth and feed utilization under the animal protein diet during the 4-week trial. DMPT supplementation significantly increased FI compared with the PPD group, although WGR, SGR, and FCR were not significantly different from either the PPD or APD group. These findings indicate that DMPT mainly exerted a feed-stimulatory effect during the short-term feeding period, while its long-term effects on growth performance and feed efficiency require further verification.

Muscle composition is a key economic trait in aquaculture species, reflecting the nutritional quality and market value of the product. In the present study, no significant differences were observed in muscle moisture, crude protein, crude lipid, or ash content among the three treatment groups. This indicates that under isonitrogenous and isolipidic conditions, all-plant protein diets can effectively replace animal protein diets without adversely affecting the accumulation of major muscle nutrients in narrow-clawed crayfish. This result is consistent with findings in Pacific white shrimp [31], gibel carp (*Carassius auratus gibelio*) [32], and silvery-black porgy (*Sparidentex hasta*) [33], confirming that plant protein ingredients can meet the nutritional requirements for muscle growth when formulated with balanced amino acids. Notably, DMPT supplementation did not exert a significant regulatory effect on muscle nutrient accumulation.

Plenty of studies have demonstrated that the hepatopancreas is the central organ responsible for nutrient metabolism and antioxidant protection in crustaceans. It also plays a key part in scavenging reactive oxygen species (ROS) and defending against lipid peroxidation [34,35,36]. Produced ultimately from ROS-mediated lipid peroxidation, MDA acts as an indirect indicator of cellular and tissue damage severity [37,38]. As important biochemical markers, T-AOC, GSH, SOD and CAT can represent the overall antioxidant status of cultured aquatic animals [39,40,41]. In the current trial, MDA contents showed no significant intergroup differences among the three treatments. This result demonstrates that both the plant protein diet and the plant protein diet supplemented with 0.5% DMPT failed to induce oxidative injury in crayfish hepatopancreas. Notably, dietary DMPT supplementation significantly increased GSH content, CAT, T-AOC, and SOD activity in the hepatopancreas of crayfish. These results suggest that DMPT did not merely prevent oxidative damage, but further strengthened the antioxidant defense system under plant protein-based feeding conditions. Previous studies have confirmed that DMPT can significantly enhance the antioxidant capacity of marine algae by scavenging harmful hydroxyl radicals [42]. Similarly, Liu et al. [18] found that DMPT could improve the activities of antioxidant enzymes in the muscle tissue of grass carp. A separate report revealed that DMPT enhances the antioxidant potential of grass carp and abalone through the activation of the Keap1/Nrf2 signaling pathway [13,17]. Collectively, these studies demonstrate that the function of DMPT is not limited to feeding stimulation but also can enhance the antioxidant defense capacity of farmed aquatic animals to a certain extent.

Intestinal morphology and digestive enzyme activities are key indicators reflecting the nutritional digestive function and health status of aquatic animals [23,43]. Histological examinations in the present study revealed that the intestinal morphology of crayfish in all three groups was well-preserved, with intact and regularly arranged intestinal villi, and no signs of epithelial damage, inflammatory infiltration, or mucosal destruction. These results indicate that neither the replacement of animal protein with plant protein nor the supplementation of DMPT caused pathological damage to the intestinal tissues of crayfish. This finding is consistent with previous studies; for instance, in Atlantic salmon (*Salmo salar*), an 80% plant protein diet did not induce intestinal enteritis [44]. Similarly, it has been reported that when the feed formula is properly optimized, a balanced mixed plant protein diet can maintain the normal intestinal morphology of Nile tilapia (*Oreochromis niloticus*) [45]. However, previous studies have also pointed out that high dietary inclusion of plant proteins can cause pathological damage to the midgut tissue of pacific white shrimp [46]. The discrepancy may be attributed to differences in ingredient quality, processing technology, and feed formulation. In the present study, high-quality plant protein ingredients such as soy protein concentrate were used, which reduced the adverse effects of anti-nutritional factors. These results suggest that under the premise of reasonably controlling the proportion of plant protein, ensuring raw material quality, and maintaining balanced feed nutrition, plant protein substitution for animal protein will not damage the intestines of aquatic animals. Furthermore, DMPT supplementation did not exert any adverse effects on intestinal structure, indicating its safety as a functional feed additive. Digestive enzyme activities directly affect the digestion and utilization of nutrients by aquatic animals [5,47]. A study on red claw crayfish (*Cherax quadricarinatus*) found that replacing 50% of fishmeal with plant protein significantly reduced the activities of trypsin and lipase [48]. On the contrary, adding DMPT to feed can markedly elevate the activities of intestinal digestive enzymes in grass carp [13] and largemouth bass fry [14]. Excitingly, consistent with these findings, both the animal protein diet group and the DMPT-supplemented group in the present study significantly enhanced the intestinal digestive enzyme activities of crayfish. Animal protein sources generally possess characteristics of high digestibility, balanced amino acid composition, and low anti-nutritional factor content, which can promote the secretion of digestive enzymes and enhance nutrient utilization efficiency [49,50,51]. As a functional feed additive, DMPT may indirectly exert its effects by promoting feed intake and improving feeding behavior, thereby enhancing intestinal digestive activity and digestive enzyme secretion capacity [14,18]. Additionally, studies have shown that DMPT can regulate neuroendocrine processes related to appetite, which may further promote digestive metabolism [17,52]. Moreover, the improvement in digestive enzyme activities may also be associated with changes in the intestinal microbial community structure, as alterations in gut microbiota can similarly influence the digestive process and nutrient metabolism of the host [53,54]. Thus, the increased feed intake, enhanced digestive enzyme activities, and improved antioxidant indices observed in the DMPT group may represent interconnected physiological responses rather than isolated effects.

The intestinal microbiota is a critical interface modulating nutrient metabolism, mucosal barrier integrity, and systemic health in aquatic animals [55]. Beyond digestion, these communities drive host physiological homeostasis through bioactive metabolite production and immunomodulatory signaling [56,57]. In the present study, β-diversity analysis revealed tighter clustering of samples in the DMPT group, indicating that DMPT supplementation promotes a more stable intestinal microbial structure in crayfish. While the APD group had markedly higher Chao1 and Pd_faith metrics than the PPD group according to α-diversity analysis, the DMPT and APD groups showed no statistically distinct changes. This suggests that DMPT supplementation effectively enhances the richness and diversity of the intestinal microbiota under plant protein-based feeding regimes. Further taxonomic profiling showed that Pseudomonadota, Bacillota and Bacteroidota dominated the core microbial community of crayfish, jointly representing over 90% of the total sequence abundance. This composition is consistent with previous reports on red swamp crayfish [58] and red claw crayfish [59]. Differential analysis further indicated that while Pseudomonadota abundance was highest in the PPD group and lowest in the APD group, the differences were not statistically significant. Notably, DMPT supplementation significantly increased the relative abundance of Bacillota. By contrast, Bacteroidota was significantly more abundant in the APD group than in the DMPT group, with its abundance being similar to that in the PPD group. Previous research has established that Bacillota and Bacteroidota play essential roles in nutrient metabolism, intestinal barrier maintenance, and immunomodulation [60,61]. In contrast, an expansion of Pseudomonadota is often regarded as a signature of dysbiosis and pathogen intrusion [62,63]. Specifically, certain Bacteroidota species produce functional metabolites that induce autoimmune responses in crustaceans [64,65], while endospores produced by Bacillota provide protective effects for crustaceans exposed to fluctuating water quality [66]. Therefore, we hypothesize that the enhanced antioxidant capacity observed in the APD and DMPT groups may be intrinsically linked to the high abundance of these two phyla in the intestine.

At the genus level, a noteworthy observation was the enrichment of *Candidatus Hepatoplasma*—a Mollicutes-related symbiont predominantly reported in isopods and specific crustaceans [67,68]. This taxon showed markedly higher abundance in the DMPT group relative to the PPD and APD groups. Studies on isopods suggest that individuals harboring this symbiont exhibit better survival rates when consuming low-quality diets [67]. Genomic analyses further suggest that *Candidatus Hepatoplasma* may provide a “defensive symbiosis” by colonizing the digestive tract surface and competing spatially with pathogens, thereby reducing infection risks in terrestrial isopods [69]. Although its ecological role in the intestine of fish and shrimp remains poorly understood, our results suggest that DMPT may facilitate the colonization and enrichment of *Candidatus Hepatoplasma* within the crayfish intestine. This shift in community structure likely contributes to the improvement of the host’s physiological status, particularly regarding antioxidant defenses, though this remains to be verified by further experimental evidence. LEfSe analysis identified specific microbial biomarkers for each treatment, suggesting that both protein source and DMPT supplementation create distinct ecological niches within the intestine. Intestinal microbial network interaction analysis showed that while positive correlations dominated all groups, the PPD vs. DMPT comparison exhibited a relatively higher proportion of negative correlations. This indicates that DMPT enhances competitive interactions within the intestinal microbiota. According to ecological theory [70], increased competition can reduce interspecies dependency and buffer the propagation of perturbations, thereby enhancing community stability. In conclusion, DMPT supplementation appears to optimize the intestinal micro-ecosystem by modulating symbiotic and competitive relationships, ultimately improving microbial stability and functional diversity.

It should be noted that this study was a short-term functional feeding trial rather than a full-cycle growth evaluation. The 4-week period allowed us to assess the rapid effects of DMPT on feed intake, digestive enzymes, antioxidant capacity, and intestinal microbiota, but was insufficient to evaluate long-term growth, feed efficiency, molting, reproduction, disease resistance, and production performance. Moreover, because DMPT was tested under voluntary feeding conditions, its direct physiological effects could not be fully separated from the indirect effects of increased feed intake. Future studies should include longer feeding periods, comprehensive growth and feed-efficiency assessments, and feed-restricted or pair-fed DMPT-supplemented controls.

## 5. Conclusions

In conclusion, dietary DMPT supplementation partially alleviates the limitations associated with plant protein diets in narrow-clawed crayfish. DMPT significantly increased feed intake and maintained growth-related indices, feed conversion ratio, and survival without adverse effects during the short-term feeding trial. In addition, DMPT enhanced digestive enzyme activities and improved antioxidant capacity in the hepatopancreas. DMPT also modulated the intestinal microbiota by reshaping community structure, increasing microbial diversity, and strengthening network stability. These findings suggest that DMPT functions as a multifunctional feed additive linking feeding stimulation, antioxidant regulation, and gut microbial homeostasis, thereby supporting the more effective replacement of animal protein with plant protein in crayfish aquafeeds.

## Figures and Tables

**Table 1:** Formulations and nutrition compositions of experimental feeds.

Items	PPD	APD	DMPT
Feed ingredients (%, dry matter)			
Soybean meal	15.00	0.00	15.00
Corn gluten meal	25.00	0.00	25.00
Soy protein concentrate	15.00	0.00	15.00
Fishmeal	0.00	25.00	0.00
Krill meal	0.00	15.00	0.00
Poultry by-product meal	0.00	10.00	0.00
High-gluten wheat flour	24.00	26.00	24.00
Soybean oil	4.00	4.00	4.00
Phospholipid oil	5.00	3.00	5.00
Vitamin premix ^1^	1.00	1.00	1.00
Mineral premix ^2^	1.00	1.00	1.00
Monocalcium phosphate	2.00	2.00	2.00
Lysine	0.70	0.00	0.70
Methionine	0.20	0.15	0.20
Threonine	0.30	0.30	0.30
Choline chloride	0.50	0.50	0.50
DMPT	0.00	0.00	0.50
Salt	1.00	1.00	1.00
Vitamin C ester	0.02	0.02	0.02
Chitinase	0.20	0.20	0.20
Microcrystalline cellulose	3.48	9.23	2.98
Zeolite powder	1.60	1.60	1.60
Total	100.00	100.00	100.00
Nutrition analysis, % dry matter basis premix ^3^			
Moisture	2.66 ± 0.11	2.45 ± 0.14	3.08 ± 0.03
Crude protein	35.88 ± 0.34	35.96 ± 0.19	35.84 ± 0.27
Crude lipid	13.14 ± 0.15	13.63 ± 0.18	13.62 ± 0.17
Ash	8.24 ± 0.29	14.51 ± 0.11	8.22 ± 0.24
GE (kJ/g diet) ^4^	21.01	20.05	21.12
NPE (kJ/g diet) ^4^	12.54	11.56	12.66

^1^ Vitamins premix (mg/kg): vitamin A, 25,000 IU; vitamin D_3_, 20,000 IU; vitamin E, 200 mg; vitamin K_3_, 20 mg; thiamin, 40 mg; riboflavin, 50 mg; calcium pantothenate, 100 mg; pyridoxine HCl, 40 mg; cyanocobalamin, 0.2 mg; biotin, 6 mg; folic acid, 20 mg; niacin, 200 mg; inositol, 1000 mg; vitamin C, 2000 mg; choline, 2000 mg. ^2^ Mixed minerals (mg/kg): calcium biphosphate, 20 g; sodium chloride, 2.6 g; potassium chloride, 5 g; magnesium sulphate, 2 g; ferrous sulphate, 0.9 g; zinc sulphate, 0.06 g; cupric sulphate, 0.02 g; manganese sulphate, 0.03 g; sodium selenate, 0.02 g; cobalt chloride, 0.05 g; potassium iodide, 0.004 g. ^3^ All were measured values. All data presented as means ± SEM (*n* = 3). ^4^ NFE, nitrogen-free extract; GE, gross energy; NPE, non-protein energy. NFE, GE, and NPE were calculated based on the mean values of crude protein, crude lipid, and ash. NFE (%) = 100 − crude protein (%) − crude lipid (%) − ash (%). GE (kJ/g diet) = [crude protein (%) × 23.6 + crude lipid (%) × 39.5 + NFE (%) × 17.2]/100. NPE (kJ/g diet) = [crude lipid (%) × 39.5 + NFE (%) × 17.2]/100.

## Data Availability

Data will be made available on request. The raw sequencing data presented in this study are openly available in [NCBl Sequence Read Archive (SRA)] [https://www.ncbi.nlm.nih.gov/bioproject/PRJNA1450897/] [PRJNA1450897] (accessed on 9 Apri 2026).

## References

[1] Harlioğlu M.M. (2004). The present situation of freshwater crayfish, *Astacus leptodactylus* (Eschscholtz, 1823) in Turkey. Aquaculture.

[2] Cai H., Chen H., Zhou H., Xu X., Guan W., Shentu X., Lou B. (2025). Characterization and phylogenetic analysis of the complete mitochondrial genome of invasive *Pontastacus leptodactylus* in China. Aquac. Rep..

[3] Chen S., Lin Y., Miao L., Liu B., Ge X. (2022). Iron homeostasis of liver-gut axis alleviates inflammation caused by dietary cottonseed meal through dietary Fe^2+^ supplementation in juvenile grass carp (*Ctenopharyngodon idellus*). Aquaculture.

[4] Chen S., Lin Y., Shi H., Miao L., Liu B., Ge X. (2022). Dietary ferulic acid supplementation improved cottonseed meal-based diet utilization by enhancing intestinal physical barrier function and liver antioxidant capacity in grass carp (*Ctenopharyngodon Idellus*). Front. Physiol..

[5] Chen S., Maulu S., Wang J., Xie X., Liang X., Wang H., Wang J., Xue M. (2024). The application of protease in aquaculture: Prospects for enhancing the aquafeed industry. Anim. Nutr..

[6] Biswas A., Araki H., Sakata T., Nakamori T., Takii K. (2019). Optimum fish meal replacement by soy protein concentrate from soymilk and phytase supplementation in diet of red sea bream, *Pagrus major*. Aquaculture.

[7] Freitas L.E.L., Nunes A.J.P., do Carmo Sá M.V. (2011). Growth and feeding responses of the mutton snapper, *Lutjanus analis* (Cuvier 1828), fed on diets with soy protein concentrate in replacement of Anchovy fish meal. Aquac. Res..

[8] Martinat M., Varvarais A., Heraud C., Surget A., Lanuque A., Terrier F., Roy J. (2025). Effects of a plant-based diet during the first month of feeding on alevin rainbow trout (*Oncorhynchus mykiss*) in the development of tongue sensory system regulating feeding behavior. Aquac. Nutr..

[9] Rhodes M.A., Zhou Y., Salze G.P., Hanson T.R., Davis D.A. (2017). Development of plant-based diets and the evaluation of dietary attractants for juvenile Florida pompano, *Trachinotus carolinus* L.. Aquac. Nutr..

[10] Hussein E.E., Habiba M.M., Ashry A.M., Al-Zayat A.M., Teiba I.I., Shehata A.I., Shahin S.A., El-Ratel I.T., Mzengereza K., Tembo M. (2023). Effects of dietary supplementation with organic acids mixture on growth, feed efficiency, hematobiochemical parameters, immunity, and intestinal microbiota of Gilthead seabream (*Sparus aurata*) juveniles. Aquac. Rep..

[11] Kattakdad S., Phungam N., Pongket U., Muangmala W., Udduang S., Aripin S.A., Yuangsoi B. (2025). Microencapsulated inulin as a prebiotic: Enhancing growth, digestive enzyme activity, and immune response in striped catfish (*Pangasianodon hypophthalmus*). Aquac. Rep..

[12] Rufchaei R., Hoseinifar S.H., Nedaei S., Bagheri T., Ashouri G., Van Doan H. (2019). Non-specific immune responses, stress resistance and growth performance of Caspian roach (*Rutilus caspicus*) fed diet supplemented with earthworm (*Eisenia foetida*) extract. Aquaculture.

[13] Liu X., Feng L., Jiang W.-D., Wu P., Jiang J., Tang L., Kuang S.-Y., Zhou X.-Q., Liu Y. (2019). Dimethyl-β-propiothetine (DMPT) supplementation under the all-plant protein diet enhances growth performance, digestive capacity and intestinal structural integrity for on-growing grass carp (*Ctenopharyngodon idella*). Aquaculture.

[14] Yang J., Wang H., Fan X., Wang J., Zhao J., Xu Q. (2025). Effects of attractants on the growth performance, antioxidant capacity, immunity, and histology of largemouth bass larvae (*Micropterus salmoides*). Aquac. Nutr..

[15] Zhou Z., Wang L., Dai M., Gao Q., Wang P., Zhao L., Li Y., Xi R., Pan M., Ma Q. (2023). Three types of enteromorpha prolifera bio-products based on different processing procedures as feed additives in the diets of pacific white shrimp (*Litopenaeus vannamei*). Fishes.

[16] Nakajima K. (1991). Effects of diet-supplemented dimethyl-β-propiothetin on growth and thrust power of goldfish, carp, and red sea bream. Nippon Suisan Gakkaishi.

[17] Liu Y., Wu Z., Yu X., Chen P., Liu Y., Han Z., Zhang Z., Fang T., Mai K., Zhang W. (2025). Dietary dimethyl-β-propiothetine supplementation for abalone *Haliotis discus hannai*: Effects on growth, digestion, immunity and appetite. Aquac. Int..

[18] Liu X.W., Feng L., Jiang W.D., Wu P., Jiang J., Yang D.M., Tang L., Kuang S.Y., Shi H.Q., Zhou X.Q. (2020). (2-Carboxyethyl)dimethylsulfonium Bromide (Br-DMPT) improves muscle flesh quality and antioxidant status of on-growing grass carp (*Ctenopharyngodon idella*) fed non-fish meal diets. Aquaculture.

[19] Zhou K.M., Liu P.P., Yao J.Y., Vasta G.R., Wang J.X., Wang X.W. (2025). Shrimp intestinal microbiota homeostasis: Dynamic interplay between the microbiota and host immunity. Rev. Aquac..

[20] Wang H., Liu P.P., Wei Z., Wang X.W. (2023). PcEiger links the Imd/Relish pathway to ROS production in the intestine of the red swamp crayfish. EMBO Rep..

[21] Ma G., Song L., Zhang Y., Zheng X., Mao S., Wang B., Xu P., Wu J., Liu B., Zhang W. (2025). Comparative analysis of growth traits, textural attributes, and blood biochemical parameters in *Procambarus clarkii* under varied feeding regimens. Isr. J. Aquac.-Bamidgeh.

[22] Latimer G.W. (2023). Official Methods of Analysis of AOAC INTERNATIONAL.

[23] Yan Y., Tang Y., Chen X., Chen X., Zhang M., Feng D., Li M. (2025). Rosiglitazone ameliorates adverse effects of high-fat diet in largemouth bass (*Micropterus salmoides*): Modulation of lipid metabolism, antioxidant capacity, inflammatory response, and gut microbiota. Antioxidants.

[24] Sánchez-Muros M.J., Renteria P., Vizcaino A., Barroso F.G. (2020). Innovative protein sources in shrimp (*Litopenaeus vannamei*) feeding. Rev. Aquac..

[25] Glencross B.D., Baily J., Berntssen M.H.G., Hardy R., MacKenzie S., Tocher D.R. (2020). Risk assessment of the use of alternative animal and plant raw material resources in aquaculture feeds. Rev. Aquac..

[26] Li X., Song C., Xie S., Wan M., Liu Y., Wang C., Su G., Huang F., Tian J. (2026). Dietary creatine supplementation promoted growth performance, muscle growth and protein deposition in red swamp crayfish (*Procambarus clarkii*) fed with all-plant-protein diets. Aquaculture.

[27] Ma S., Xiao P., Wu Z., Guo Y., Mai K., Zhang W. (2025). Multi-omics reveal the effect of different dietary plant protein sources on the microbiota-gut-digestive gland axis of abalone *Haliotis dicus hannai*. Aquaculture.

[28] Willora F.P., Vatsos I.N., Mallioris P., Bordignon F., Keizer S., Martınez-Llorens S., Sørensen M., Hagen Ø. (2022). Replacement of fishmeal with plant protein in the diets of juvenile lumpfish (*Cyclopterus lumpus*, L. 1758): Effects on digestive enzymes and microscopic structure of the digestive tract. Aquaculture.

[29] Hu J., Le Q., Wang Y., Kuang S., Zhang M., Gu W., Sun Y., Jacques K.J., Li Y., Zhang Y. (2020). Comparative transcriptome analysis of olfactory epithelium in large yellow croaker: Evidence for olfactory adaptation to feed phagostimulant in fish. Aquaculture.

[30] Nakajima K., Kiene R.P., Visscher P.T., Keller M.D., Kirst G.O. (1996). Effects of DMSP and Related Compounds on Behavior, Growth and Stress Resistance of Fish, Amphibians and Crustaceans. Biological and Environmental Chemistry of DMSP and Related Sulfonium Compounds.

[31] Zhao R., Wang X., Zhou H., Liu C., Mai K., He G. (2025). Replacing fishmeal with composite plant protein meal affects nutrient metabolism via mTOR signaling pathway of Pacific white shrimp (*Litopenaeus vannamei*). Anim. Nutr..

[32] Cai W., Liu H., Han D., Zhu X., Jin J., Yang Y., Xie S. (2022). Complete replacement of fishmeal with plant protein ingredients in gibel carp (*Carassius auratus gibelio*) diets by supplementation with essential amino acids without negative impact on growth performance and muscle growth-related biomarkers. Front. Mar. Sci..

[33] Yaghoubi M., Mozanzadeh M.T., Marammazi J.G., Safari O., Gisbert E. (2016). Dietary replacement of fish meal by soy products (soybean meal and isolated soy protein) in silvery-black porgy juveniles (*Sparidentex hasta*). Aquaculture.

[34] Yan Y., Lin Y., Zhang L., Gao G., Chen S., Chi C., Hu S., Sang Y., Chu X., Zhou Q. (2023). Dietary supplementation with fermented antarctic krill shell improved the growth performance, digestive and antioxidant capability of *Macrobrachium nipponense*. Aquac. Rep..

[35] Duan Y., Zhong G., Xiao M., Yang Y., Wang Y., Nan Y. (2024). Integrated physiological, energy metabolism, and metabonomic responses indicate the stress response in the hepatopancreas of *Litopenaeus vannamei* to nitrite stress. Aquat. Toxicol..

[36] Han S.Y., Wang M.Q., Wang B.J., Liu M., Jiang K.Y., Wang L. (2018). A comparative study on oxidative stress response in the hepatopancreas and midgut of the white shrimp *Litopenaeus vannamei* under gradual changes to low or high pH environment. Fish Shellfish Immunol..

[37] Yan Y., Lin Y., Gu Z., Lu S., Zhou Q., Zhao Y., Liu B., Miao L. (2024). Dietary fishmeal substitution with Antarctic krill meal improves the growth performance, lipid metabolism, and health status of oriental river prawn (*Macrobrachium nipponense*). Aquac. Rep..

[38] Zhu W., Li Q., Peng M., Yang C., Chen X., Feng P., Liu Q., Zhang B., Zeng D., Zhao Y. (2024). Biochemical indicators, cell apoptosis, and metabolomic analyses of the low-temperature stress response and cold tolerance mechanisms in *Litopenaeus vannamei*. Sci. Rep..

[39] Chen S., Lin Y., Miao L., Pan W., Jiang W., Qian L., Hao J., Xi B., Liu B., Ge X. (2021). Ferulic acid alleviates lipopolysaccharide-induced acute liver injury in *Megalobrama amblycephala*. Aquaculture.

[40] Li J.T., Zhao Y.H., Lv Y., Su X., Mei W.L., Lu Y.P., Zheng P.H., Zhang Z.L., Zhang X.X., Chen H.Q. (2023). Evaluating the antioxidant properties of the leaves and stems of *Alpinia oxyphylla* in vitro and its growth-promoting, muscle composition change, and antioxidative stress function on juvenile *Litopenaeus vannamei*. Antioxidants.

[41] Li Y., Chen J., Jiang S., Huang J., Jiang S., Yang Q., Yang L., Shi J., Zhou F. (2024). A comprehensive study on nutritional quality, physiological enzyme activity and genetic diversity in six populations of *Penaeus monodon*. Aquac. Int..

[42] Sunda W., Kieber D.J., Kiene R.P., Huntsman S. (2002). An antioxidant function for DMSP and DMS in marine algae. Nature.

[43] Chen S., Liang X., Wang J., Wang H., Zhu Y., Li C., Ma S., Wang J., Xue M. (2026). Hydrolysate of a low-carbon microbe protein improved the performance and antioxidant capacity in *Micropterus salmoides*. Aquac. Rep..

[44] Egerton S., Wan A., Murphy K., Collins F., Ahern G., Sugrue I., Busca K., Egan F., Muller N., Whooley J. (2020). Replacing fishmeal with plant protein in Atlantic salmon (*Salmo salar*) diets by supplementation with fish protein hydrolysate. Sci. Rep..

[45] Soltan N.M., Soaudy M.R., Abdella M.M., Hassaan M.S. (2023). Partial dietary fishmeal replacement with mixture of plant protein sources supplemented with exogenous enzymes modify growth performance, digestibility, intestinal morphology, haemato-biochemical and immune responses for Nile tilapia, *Oreochromis niloticus*. Anim. Feed Sci. Technol..

[46] Kasamechotchung C., Munkongwongsiri N., Plaipetch P., Lertsiri K., Thitamadee S., Vanichviriyakit R., Khidprasert S., Sritunyalucksana K., Façanha F.N., Kruangkum T. (2025). Effect of partial and total replacement of fishmeal by soybean meal in feed on growth and gut performance of *Penaeus vannamei*. Sci. Rep..

[47] Rafanan K.C., Herrera M.J., Catabay C., German D.P. (2025). Diet shifts alter the activity and distribution of digestive enzymes in an herbivorous fish. Fish Physiol. Biochem..

[48] Qian D., Yang X., Xu C., Chen C., Jia Y., Gu Z., Li E. (2021). Growth and health status of the red claw crayfish, *Cherax quadricarinatus*, fed diets with four typical plant protein sources as a replacement for fish meal. Aquac. Nutr..

[49] Hussain S.M., Bano A.A., Ali S., Rizwan M., Adrees M., Zahoor A.F., Sarker P.K., Hussain M., Arsalan M.Z.-u.-H., Yong J.W.H. (2024). Substitution of fishmeal: Highlights of potential plant protein sources for aquaculture sustainability. Heliyon.

[50] Ma Y., Su Z., Chen F., Xu C., Jiang K., An W., Zhang G., Xie D., Wang S., Dong Y. (2023). Terrestrial Compound Protein Replacing Dietary Fishmeal Improved Digestive Enzyme Activity, Immune Response, Intestinal Microflora Composition, and Protein Metabolism of Golden Pompano (*Trachinotus ovatus*). Aquac. Nutr..

[51] Wei J., Fu Y., Feng S., Zhang J., Zhang Y., Yu J., Kang P., Wu C., Mi H. (2025). The effects of fishmeal replacement with degossypolled cottonseed protein on growth, serum biochemistry, endocrine responses, lipid metabolism, and antioxidant and immune responses in black carp (*Mylopharyngodon piceus*). Animals.

[52] Li W., Li E., Wang S., Liu J., Wang M., Wang X., Qin C., Qin J., Chen L. (2025). Comparative effects of four feed attractants on growth, appetite, digestion and absorption in juvenile Chinese mitten crab (*Eriocheir sinensis*). Aquaculture.

[53] Jiao F., Zhang L., Limbu S.M., Yin H., Xie Y., Yang Z., Shang Z., Kong L., Rong H. (2023). A comparison of digestive strategies for fishes with different feeding habits: Digestive enzyme activities, intestinal morphology, and gut microbiota. Ecol. Evol..

[54] Zhang G.G., Yang Z.B., Wang Y., Yang W.R., Zhou H.J. (2014). Effects of dietary supplementation of multi-enzyme on growth performance, nutrient digestibility, small intestinal digestive enzyme activities, and large intestinal selected microbiota in weanling pigs. J. Anim. Sci..

[55] Medina-Félix D., Garibay-Valdez E., Vargas-Albores F., Martínez-Porchas M. (2023). Fish disease and intestinal microbiota: A close and indivisible relationship. Rev. Aquac..

[56] Shi Y., Liu Y., Zheng S., Dai J., Zhang J., Xu S., Zhong L., Xie S., Hu Y. (2026). Nutritional and functional enhancement of cottonseed meal via diverse processing techniques as soybean meal alternatives in grass carp (*Ctenopharyngodon idella*): Impacts on growth, muscle texture, and intestinal homeostasis. Anim. Nutr..

[57] Wang S., Li X., Zhang M., Li M. (2026). Dietary sodium propionate optimizes intestinal microbiota and promotes growth performance, lipid metabolism and ammonia tolerance in yellow catfish (*Pelteobagrus fulvidraco*). Anim. Nutr..

[58] Xie M., Zhang S., Xu L., Wu Z., Yuan J., Chen X. (2021). Comparison of the intestinal microbiota during the different growth stages of red swamp crayfish (*Procambarus clarkii*). Front. Microbiol..

[59] Li W.F., Zhao A.Q., Chen Y., Yin Z.Y., Mao Y.X., Qu Z., Zhang S., Huang H. (2025). Key differences in the gut microbiota of red-claw crayfish *Cherax quadricarinatus* with different sizes and genders under consistent farming conditions. Biology.

[60] Ringø E., Harikrishnan R., Soltani M., Ghosh K. (2022). The effect of gut microbiota and probiotics on metabolism in fish and shrimp. Animals.

[61] Zheng X., Xu X., Liu M., Yang J., Yuan M., Sun C., Zhou Q., Chen J., Liu B. (2024). Bile acid and short chain fatty acid metabolism of gut microbiota mediate high-fat diet induced intestinal barrier damage in *Macrobrachium rosenbergii*. Fish Shellfish Immunol..

[62] Kim P.S., Shin N.R., Lee J.B., Kim M.S., Whon T.W., Hyun D.W., Yun J.H., Jung M.J., Kim J.Y., Bae J.W. (2021). Host habitat is the major determinant of the gut microbiome of fish. Microbiome.

[63] Shin N.R., Whon T.W., Bae J.W. (2015). *Proteobacteria*: Microbial signature of dysbiosis in gut microbiota. Trends Biotechnol..

[64] Fan L., Li Q.X. (2019). Characteristics of intestinal microbiota in the Pacific white shrimp *Litopenaeus vannamei* differing growth performances in the marine cultured environment. Aquaculture.

[65] Zhang S., Sun X. (2022). Core gut microbiota of shrimp function as a regulator to maintain immune homeostasis in response to wssv infection. Microbiol. Spectr..

[66] Ding Y., Sun Y., Cheng Y. (2025). Effects of different feeding modes on the growth, reproduction, digestion, stress tolerance, and intestinal microflora of pre-adult red swamp crayfish *Procambarus clarkii*. Aquaculture.

[67] Fraune S., Zimmer M. (2008). Host-specificity of environmentally transmitted *Mycoplasma*-like isopod symbionts. Environ. Microbiol..

[68] Leclercq S., Dittmer J., Bouchon D., Cordaux R. (2014). Phylogenomics of “*Candidatus* Hepatoplasma crinochetorum,” a Lineage of *Mollicutes* Associated with Noninsect Arthropods. Genome Biol. Evol..

[69] Kawato S., Nozaki R., Kondo H., Hirono I. (2024). Metagenome-assembled genomes of three *Hepatoplasmataceae* provide insights into isopod-mollicute symbiosis. Access Microbiol..

[70] Coyte K.Z., Schluter J., Foster K.R. (2015). The ecology of the microbiome: Networks, competition, and stability. Science.

